# Critical Design Considerations for Longer-Term Wear and Comfort of On-Body Medical Devices

**DOI:** 10.3390/bioengineering11111058

**Published:** 2024-10-24

**Authors:** Shavini Stuart, Margreet de Kok, Ben O’Searcoid, Hannah Morrisroe, Irina Bianca Serban, Ferry Jagers, Remon Dulos, Steven Houben, Linda van de Peppel, Jeroen van den Brand

**Affiliations:** 1 TNO, Holst Centre, High Tech Campus 31, 5656AE Eindhoven, The Netherlands; 2 Industrial Design and Artificial Intelligence Systems Institute, Eindhoven University of Technology, 5612AZ Eindhoven, The Netherlands; 3 TNO, Healthy Living, Sylviusweg 71, 2333BE Leiden, The Netherlands

**Keywords:** non-woven tapes, skin-based wearable devices, user perception, higher activity, MVTR, wear and comfort

## Abstract

The commercialization of a growing number of wearable devices has been enabled within recent years due to the availability of miniaturized sensor modalities, the development of new materials, and the scalability of flexible electronics. With the increase in resource shortages within healthcare, there is a demand to translate wearable devices from the commercial consumer stand-point to the medical field. Clinical-grade signal quality, wearability, and comfort all need to be tailored to a wearable design. Wear and comfort for user compliance and durability for longer-term use are commonly overlooked. In this study, the relationship of on-body location and material layer composition is investigated. Five non-woven medical tapes noted for longer wear time are tested over a 7-day timeframe. The impact of material properties, such as elasticity, isotropy, and hysteresis, as well as the moisture vapor transmission rate (MVTR) and adhesive thickness, are evaluated in relation to skin properties on the lower torso of 30, high-activity-level volunteers. User perception was quantified via Likert-scale questionnaires and images were obtained for the material–skin interaction. The results indicate that critical characteristics, such as MVTR and elasticity, noted for positive skin interaction in commercial products, may not translate to improved user perception and durability over time. Future work will assess new design options to manipulate material properties for improved wear and comfort.

## 1. Introduction

There is a growing number of electronic wearable devices commercialized within the consumer domain [1]. The increased functionality of these devices for physiological monitoring and improved user uptake have paved the way for the needed development step for wearable digital tools within healthcare. The commercialization of clinical-grade wearable devices for healthcare applications is still in its infancy, although it is possible with sensor miniaturization and conformable flexible electronics [2]. Clinical confidence for sensor performance [3] and reduced motion artifacts with longer wear times in home settings [4] are key for the integration of point-of-care medical wearable devices. 

Recent advancements in wearable devices have focused primarily on sensor functionality and integration in proof-of concept settings [5]. There are a large number of parameters for on-skin devices that can be evaluated, such as sweat, body temperature, oxygen saturation, movement, and electrophysiological signals. These can be quantified digitally through the use of optical, electrical, electromechanical, or electrochemical signal transduction [6]. Each sensor modality has individual challenges that need to be overcome for an accurate clinical signal. In academia, new material developments for conformable, miniaturized, higher-sensitivity sensors are commonly evaluated, such as the incorporation of graphene-based sensors [7], chromic material [8], and semiconductor components [9]. New substrates are also under development for the scalable deposition and bonding of sensors in flexible circuits. Typically, this is achieved through the use of tunable polymeric materials, such as polyethylene terephthalate (PET), polyethylene naphthalate (PEN), polyimide (PI), and, increasingly, elastomeric thermoplastic polyurethane (TPU) [10]. 

There is a technology pull for the application of multiple sensor modalities to a single wearable device for multi-vital sign monitoring. This is challenging due to the material build-ups required, such as for optical uses in comparison to electrochemical uses, but also the form factor and location in which the wearable device is placed. Signal resolution needs to match sensor placement on the body. There are new development directions for larger-area wearable devices as current form factors do not meet use-case demands. Some examples include the use of single-lead wearable devices in comparison to the gold-standard 12-lead systems for electrocardiography (ECG) [11]. The current limitation in the application of larger wearable patches is that there is a minimal understanding of skin wearable devices regarding user perception, compliance, comfort, and wearability. 

Different form factors are now present for wearable devices, from generally known smartwatches to research-grade wearable patches [5]. For medical wearables required for continuous monitoring, the use of wearable patch devices are more advantageous than removable, hard metal, or plastic form factors. The movement towards more conformable wearable devices that enable a higher sensor–skin interaction is a needed development step for the creation of electronic skins (e-skins). This concept has been heralded as the future of wearable devices and, as such, is a significant step to understanding user compliance with clinical-grade signal possibilities of wearable-skin designs. A wearable patch enables improved sensor–skin interaction and, as such, improved continuous signal quality in comparison to smartwatches. There are increased possibilities within the design to reduce motion artifacts with movement with wearable patch form factors [12] and also for more freedom in the placement on the body. The limitations, however, are the interaction of the wearable patch with the skin and detrimental effects with increased time. Skin irritation caused by the reduced breathability, design, and material selection of the overall device is often overlooked but leads to reduced user compliance and detrimental impacts on vulnerable skin [13]. Smart watches, in comparison, due to their removable form factor, can be worn from months to years but are restricted in the placement location and signal resolution for continuous monitoring. 

For clinical-grade wearable devices, increased research into the use of wearable patches and possibilities for improved skin–patch interaction needs to be conducted more extensively. Due to the increased number of available wearable devices for monitoring, useful knowledge on comfort and user compliance is becoming more available [14]. A recent study for commercial wristband form factors showed low user compliance in terms of repeated or continuous use after 6 months [1]. Users were shown to remove the wristbands more commonly during the night and, as such, continuous monitoring within a clinical setting was limited. There was also a significant difference in the user acceptance of wearable devices dependent on the population group, with older population groups indicating digital barriers for technology acceptance [15]. A study by Yang et al. elaborated further on these challenges. The perceived benefit strongly influenced the perceived value of the wearable device and, as such, longer-term user compliance. This perceived benefit can be divided into three main categories: perceived usefulness, enjoyment, and social image [16]. For longer-term medical wearables, the application for the best signal quality may not align with the perceived enjoyment and social image and, as such, can hinder user compliance. To improve user perception for the next generation of devices, properties of wearable on-body devices being lightweight, conformable, and breathable are needed [17]. With increased opportunities for creating flexible electronics through screen printing of conductive inks onto a larger variety of biocompatible, stretchable substrates, wearable design can be improved for user compliance over larger population groups. 

Material selection, design, and interaction with skin and body movement pose a significant challenge for matching skin properties with man-made designs for the development of these e-skins [18]. 

Properties commonly taken into account for development of man-made materials is matching human skin’s mechanical and physiological characteristics. Human skin is an anisotropic, viscoelastic, multi-component structure having a Young’s Modulus within the range of 5 kPa to 140 MPa [19]. The deformation of body surfaces is dependent on body location and can range from 30% on skin to 100% or more on joints [14]. With the large degree of motion that human bodies can accomplish and constant repetitive movements, such as with daily breathing, which comprises over 20,000 breaths per day, material integrity is challenged in relation to attachment to the skin. This is further enhanced through external factors implicated within home-setting use–cases, such as humidity variations of the skin from the impact of sweating and showering, to interaction with different layers of clothing [20]. For a stable sensor–skin interface from a biological standpoint, the skin undergoes an epidermal turnover by cell renewal of the stratum corneum over 7–14 days. As well as cell renewal, the skin releases sweat for thermal regulation, which can vary depending on body location. The number of sweat glands present can range from 14–24 per cm^−2^ on the upper chest to 45–54 per cm^−2^ on the mid-forearm. Therefore, to maintain skin breathability and permeability, materials must provide adequate flow for perspiration and oxygen to the skin, which can vary significantly depending on the location, use–case, and duration of wear. If the selection of material does not match skin properties for wearable device application, the repercussions can be detrimental, such as skin irritation, which can vary in severity from contact dermatitis to maceration, resulting in reduced user compliance and quality of life post application [13].

The material selection of non-woven medical tapes, a dominant layer within wearable-skin device build-ups, is sparsely addressed within the literature for the impact it plays within clinical wearable devices. A large range of polymer composites, available within non-woven medical tape designs, commonly aim to achieve three main functions, mechanical integrity, physical inertness, and skin permeability, resulting in a stable skin–device interface [21]. To enable longer-term wear on the body, non-woven material developments have focused on mechanical and material design manipulation. The overall build-up of the polymer fibers and porosity is controlled for increased breathability and translated to moisture vapor transmission rates (MVTRs) [22]. The selection of adhesives, for longer-term wear within the medical field, has directed use of pressure-sensitive adhesives (PSAs) within wearable device settings on skin. PSAs work through viscoelastic properties of the material upon the application of force. These properties enable the adhesive to spread and conform to rough surfaces on skin, causing increased bonding to dynamic surfaces. Over time, the adhesive tack increases due to hydrogen and van der Waals bonds created between the skin surface and the adhesive. The adhesion strength is directly related to the viscoelastic internal properties of the material and the balance between interfacial adhesion and cohesive strength [23]. To enable effective wetting of a surface, cohesive strength is required for improved adhesive stability when bonding and for device movement, whereas adhesive strength is required for a ‘solid-like’ [23] contact during wear for durability. The most commonly used pressure-sensitive adhesives for longer wear times are acrylic-based, with tunable viscoelasticity from altered carbonyl functionalized chains upon a hydrocarbon backbone. Through tunable synthesis of these chains, improved water permeability and adhesive entanglement with the skin surface are possible [24]. 

The test methodologies for the material properties of these medical tapes, however, are constrained to small area-sized patches [25]. As of now, wearable devices in current configurations have not achieved continuous monitoring over 14 days [1]. Research and new commercial materials indicate possibilities for an increased duration of wear for up to 21 days, but the application of these materials whilst also maintaining the integrity of sensor–skin contact is still to be seen within a wearable device [26]. Therefore, there is a need to translate these properties into larger-area wearable designs. With multiple layers of different permeable materials present within wearable design build-ups, there is increased complexity in understanding user comfort and wearability [27].

Within this study, we aim to assess the significance of specific material properties within commercial non-woven tapes in a clinically relevant, printed, wearable design configuration. Accelerated testing on volunteers over 7 days was used to assess the integrity of wearable devices, with durability of the material, wearability, and user perception on comfort studied, using behavioral and visual tools. 

To the best of our knowledge, this is the first time that a study of commercial non-woven medical tape within a printed electronic wearable device design format was conducted in regards to the user perception of comfort and wearability, with increased time and higher activity. 

## 2. Materials and Methods

### 2.1. Study Design

**Figure 1:**
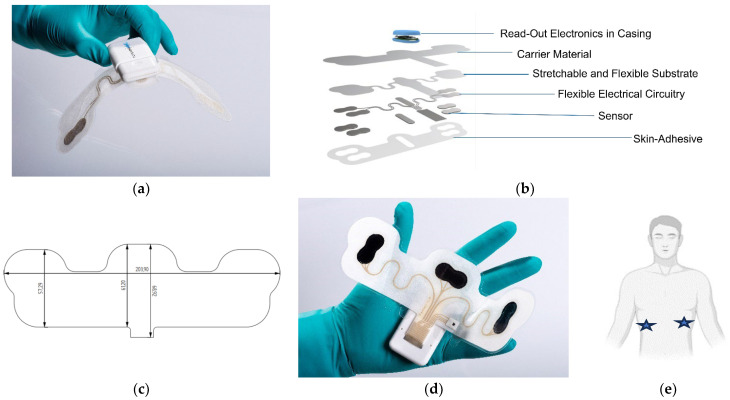
Images of the vital sign research platform: the (**a**) overall image of the wearable device; (**b**) description of the layers that comprised the vital sign research platform within the study, and the non-printed configuration consisted of the carrier material and skin adhesive, and the printed configuration consisted of all layers except read-out electronics; (**c**) dimensions of the wearable device; (**d**) wearable device showing placement of circuitry and sensors within the device; (**e**) placement indicated by blue stars for the location of wearable devices either with or without flexible circuitry incorporated on the volunteer torso’s as applied in this report.

A vital sign research platform, developed by the Holst Centre, TNO, was used to investigate the significance of non-woven tapes, defined as carrier materials and skin adhesives within the wearable device build-up. The vital sign research platform was fabricated using hybrid printed electronics, with individual layers able to be configured dependent on use–case requirements. The wearable device design was 20.3 cm by 6.8 cm in x and y directions, with curved indents within the design to improve conformability on the body. The printed ink used for the electronic design was CI-1036 Ag paste (Nagase America LLC, New York, NY, USA) screen-printed on TE-11C TPU (Dupont, Mechelen, Belgium). The design of the device was primarily focused on single-lead ECG measurements, with the surface area of the flexible electronic substrate providing a coverage of 38% to the overall design. Therefore, the dominance of the carrier material properties and influence with the flexible substrate was evaluated within two fabricated patches placed on the torso of volunteers, Figure 1. Of the two patches, one was equipped with circuitry and sensor design and called the printed patch in this report. The other contained only the carrier material and skin adhesive, called the non-printed patch. 

A 30-person volunteer study was conducted over a 7-day timeframe in collaboration with Technical University Eindhoven (TuE) (ERB2022ID54, Ethical Review Board TU/e). Volunteers were between the ages of 19 and 25 and selection criteria required participants to partake in high-intensity activity for a minimum of 2 h daily. The application and removal of the wearable devices without read-out electronics were conducted by research coordinators. If wearable patches were removed earlier within the study by volunteers or they detached due to failure in wearable-skin integrity, volunteers were asked to contact research coordinators. The location of the wearable patch was selected due to indications from cardiologists on the positioning and related lead correlation for ECG that could be of interest, V4 to V6.

#### 2.1.1. Material Selection

Within the study, 5 non-woven medical tapes with a pressure-sensitive acrylic adhesive were selected due to their commercial claims for application in wearable devices, as well as their potential for longer-term wear (14+ days) or increased user comfort. 

This was differentiated further into variations within their material properties detailed by the manufacturer, with a focus on the adhesive thickness, moisture vapor transmission rate (MVTR), and carrier material being either polyurethane (PU), polyester (PES), or polyethylene terephthalate (PET), as seen in Table 1. Commercial non-woven tapes used were DM22791, DM23171, DM45150 (Lohman-Tapes, Neuwied, Germany), and MED5740 (Avery Dennison, Cork, Ireland). The non-woven tapes were labeled from A to E, Table 1. It should be noted that with different manufacturers, the type of acrylic adhesive may vary and, as such, the permittivity with water in relation to adhesive thickness. This is visualized within Table 1, where low adhesive thickness in patch Group E did not imply higher MVTR scores; instead, MVTR scores remained within the same range as the other patch groups, which had higher adhesive thickness. 

#### 2.1.2. Wear and Comfort Assessment

Two methodologies were used during the study to obtain increased understanding on the relationship between user perception, material durability, and skin interaction with carrier material selected. A mobile phone application was created by TNO, named ‘How Am I’, as a research tool within the study. Within the 7-day timeframe, two methods of assessment—volunteer questionnaires and visual images—were used. 

Within the app, volunteers were required to firstly fill in a baseline questionnaire related to their skin perception and daily routine. The volunteer’s perception of their skin properties was then correlated to assumed physical skin properties. More showers or an increased number of toiletries could be an indication of dry skin. 

**Figure 2:**
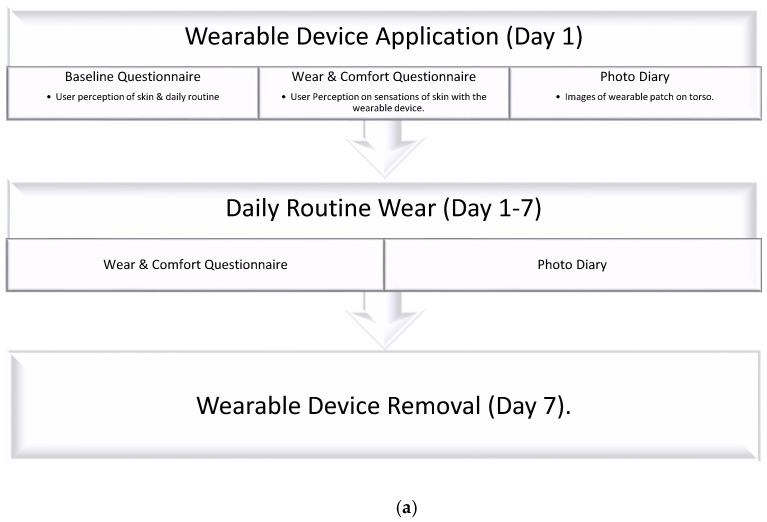

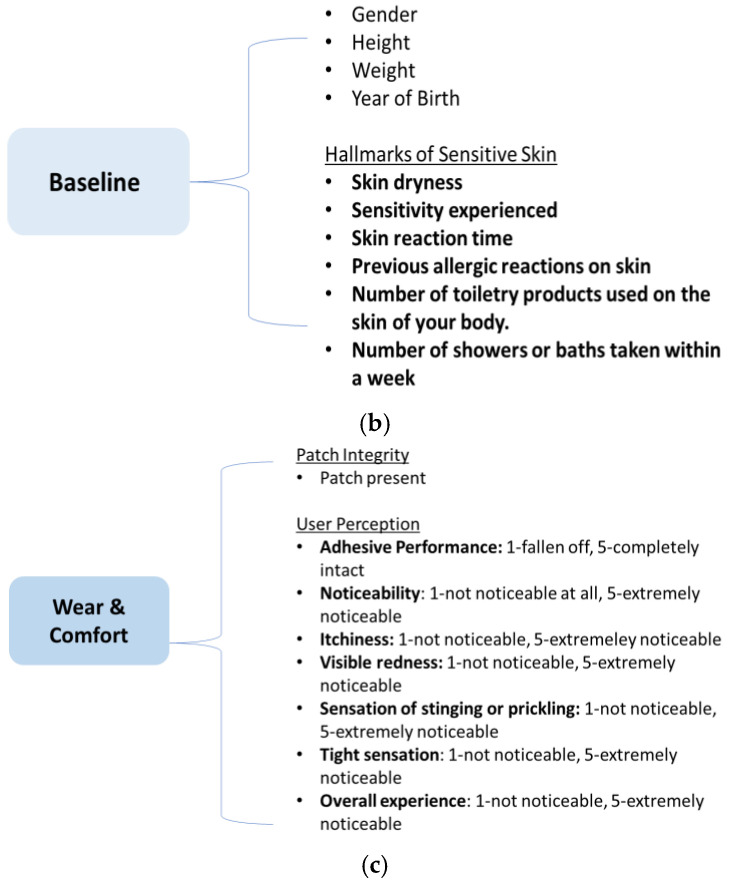
Diagrams to illustrate the (**a**) process flow of actions required by volunteers within the study, (**b**) question areas asked at the beginning of the volunteer study, and (**c**) question areas covered during the volunteer study.

Thereafter, during the 7-day timeframe, a wear and comfort questionnaire was required to be filled in for their perception on skin irritation and patch noticeability, Figure 2. Questions were asked within a Likert scale format to quantify user perception, with questions on itchiness, redness, tightness, prickling or bumps, and noticeability repeatedly asked due to their strong correlation as hallmarks of sensitivity on skin. 

At the end of the study, if the patches remained attached to the volunteers, removal was conducted by study coordinators.

### 2.2. Tensile Testing

Regarding mechanical properties, the non-woven tapes within the study were cut out of strips of 25 × 100 mm from non-woven sheets in three directions (horizontal, vertical, and diagonal) and a mechanical testing instrument (Mark-10 303-EM Motorised Force Tester, Copiague, NY, USA) was used at a 50 mm stroke, 100 mm/min, for 3 repeated cycles. The initial elastic modulus and hysteresis were then calculated.

To reduce complexity in the evaluation of wearable-skin interaction, the mechanical properties of the TPU substrate used as the substrate for printed electronics are provided below as all substrates consist of the same printed electronic layer. The material was tested mechanically in a cyclic load test for 1000 cycles at 20% strain, with a standard test strip of 76 mm for testing at 500 mm/min. The results showed that this substrate–ink combination had an initial Young’s Modulus of 33 N/m^2^ and a residual strain of 3.1% after 1000 cycles. 

### 2.3. Data Analysis

The volunteer study groups were anonymized, with random placements of patch sets, to allow a comparison between the wear and comfort properties of non-woven materials. A minimum of 5 volunteers were used within each patch group. For quantitative aspects within the volunteer study, questionnaires were prepared using 5-point Likert scales. For all quantitative methods, repeats of a minimum of 3 materials were used to evaluate trend and significance. Values exported were grouped and measured for central tendency, with standard error used. Visual grouping and image assessment were used for photo diary assessments of material durability and skin interaction. 

## 3. Results

### 3.1. Study Population

**Figure 3:**
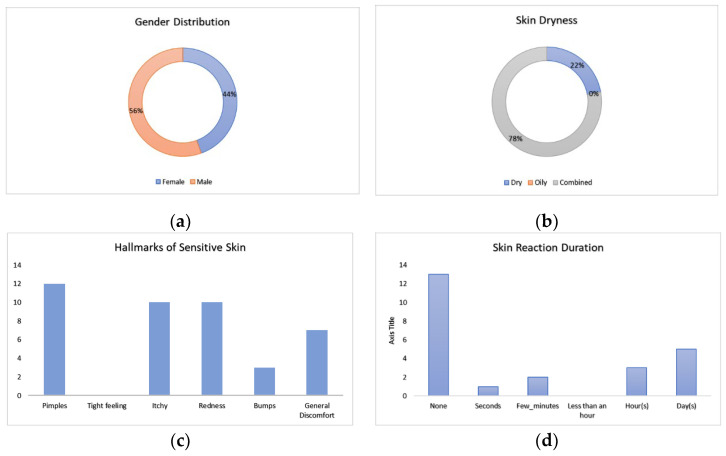
Graphs to indicate the baseline volunteer study population and previous experience with skin reactions: (**a**) the pie chart noting the gender distribution within the study; (**b**) the pie chart to note volunteer perceived skin dryness; (**c**) the bar graph to note volunteers’ perception of their skin sensitivity; and (**d**) the bar graph to note the volunteers’ perception for the duration of the skin reaction.

Volunteers that comprised the study were composed of 56% males and 44% females. Upon intake, baseline questionnaires were answered before the application of the patch configurations. The results from the baseline questionnaire indicated that volunteers did not generally view their skin as having a tendency towards either a dry or oily nature, with the majority (78%) perceiving their skin as combined. Volunteers noted a range of reactions for skin properties, with pimpling scoring the highest and tightness of skin not perceived. For the majority of volunteers, no skin reaction was previously observed, with volunteers that did notice a reaction noting it would then occur the majority of the time for days, Figure 3. These insights into skin perception are important for assessing the significant user perception of the wearable device.

### 3.2. Mechanical Properties

The non-woven material within the study was tensile-tested in three directions for mechanical properties related to elasticity, hysteresis, and isotropy.

**Figure 4:**
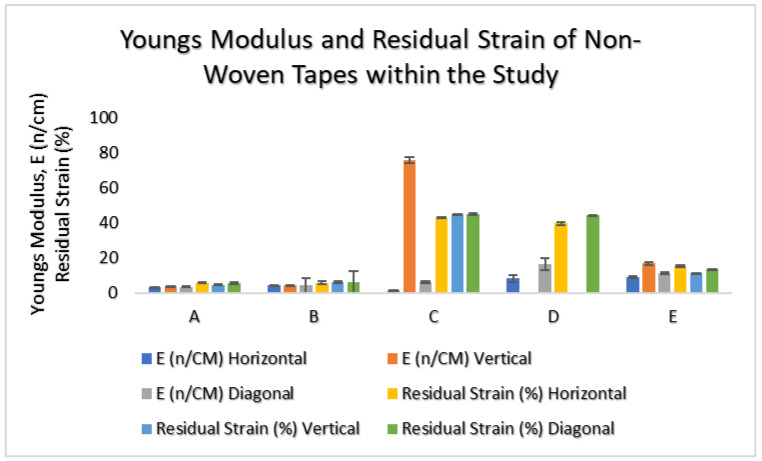
The bar graph to show the mechanical properties of non-woven tape material within the study (**A**–**E**) for Young’s Modulus and residual strain.

The results indicated that in three of the patch groups, A, B, and E, all PU-based carrier materials were isotropic, with a very low Young’s Modulus. Groups A and B had the lowest values, with low hysteresis seen by residual strains of 5–6%. For Group E, there was more hysteresis observed with a residual strain of 10–15%, Figure 4.

For Groups C and D, which comprised PES or PET, respectively, the mechanical results indicated that both materials were anisotropic. Group D was not tested for elasticity and residual strain within the vertical direction due to failure within the material during tests. For both Groups C and D, the highest elasticity was observed in the horizontal direction. Hysteresis in Group C was observed in all three directions within the range of 39–45%, whereas for Group D, residual strain was observed and similar to Group C in the horizontal and diagonal directions. 

### 3.3. Printed vs. Non-Printed Wear and Comfort

**Figure 5:**
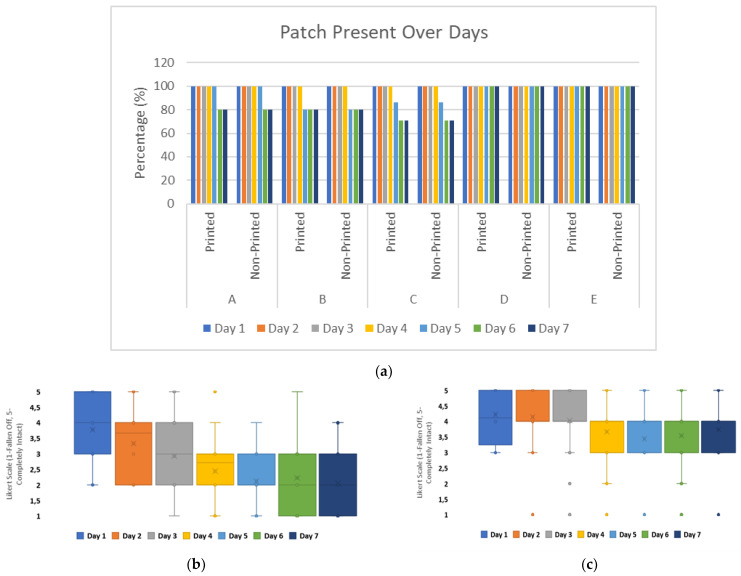
Graphs to show patch durability over 7-day timeframe: (**a**) bar graph to show patch group adherence percentage for printed and non-printed configurations on volunteers; (**b**) box plot to show user perception of adhesive performance for printed patch configurations; (**c**) box plot to show user perception of adhesive performance for non-printed patch configurations.

The durability of wearable devices over the 7-day timeframe showed that from day 5, 20% of the patches were no longer adhered for Groups A, B, and C. Group C had the most significant failure in adhesion by day 6, with a further reduction of 10% of patches still adhered. The presence of a flexible electronic substrate, underneath the carrier material, did not impact the patch integrity as results for both printed and non-printed patch configurations showed similar losses within the timeframe, Figure 5a. 

The user perception of adhesive quality for wearable patches was impacted by the presence of printed substrates underneath the carrier material. For printed patch configurations, the adhesive performance was perceived more negatively from day 2 onwards. 

For non-printed substrate configurations, the user perception on adhesive performance remained positive or neutral over the 7-day timeframe on average, with reduction in positive user perception from day 3 onwards towards neutrality, Figure 5b,c.

When comparing patch performance with material properties of the non-woven tapes, Table 1 and Figure 5, the relationship with patch groups indicates that adhesive thickness and MVTR do not fully translate to wearable device integrity over increased timeframes. Group B, which had a 20% loss in patch integrity from day 5, had the highest adhesive thickness present of the sets at 100 microns. However, Group E, which remained at 100% for patch integrity over the 7-day study, had the lowest adhesive thickness of the sets at 51 microns. Group C, which had the highest reduction in patch integrity with a 30% loss from day 6, had a 75-micron adhesive volume, similar to three other groups, and the highest MVTR, 1200 g/m^2^/h, of the sets.

**Figure 6:**
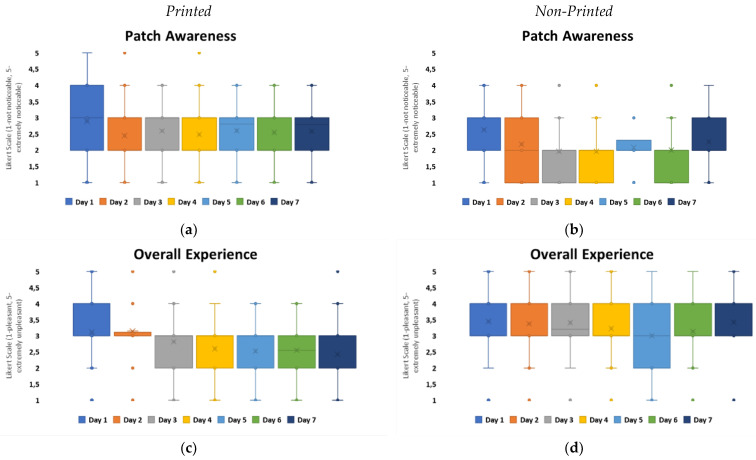
Graphs to show patch awareness and overall experience over 7-day timeframe: (**a**) box plot to show user perception of patch awareness for printed patch configurations, (**b**) box plot to show user perception of patch awareness for non-printed patch configurations, (**c**) box plot to show user perception of overall experience for printed patch configurations, (**d**) box plot to show user perception of overall experience for non-printed patch configurations.

In order to further evaluate the relationship of material properties with user perception, the awareness of the presence of the patch material and overall experience were observed as shown in Figure 6. It indicated that when comparing printed electronic and non-printed electronic configurations, volunteers were more neutral or positively biased towards printed configurations, with improvement seen and remaining constant from day 2 onwards. With non-printed versions, user perception fluctuated within awareness on day 5 and 7. 

Patch awareness, however, did not fully correlate with the overall experience of volunteers. Printed configurations remained at a neutral to positive bias for overall experience from day 2 onwards. However, for non-printed configurations, the overall experience was observed to be neutral to slightly negative, which was constant over the 7-day timeframe. These results indicate that awareness of patch material may not correlate fully with positive perceptions of wearability and comfort. Also, the aesthetics of the wearable device for volunteers may impact their perception as supported within the literature [17].

**Figure 7:**
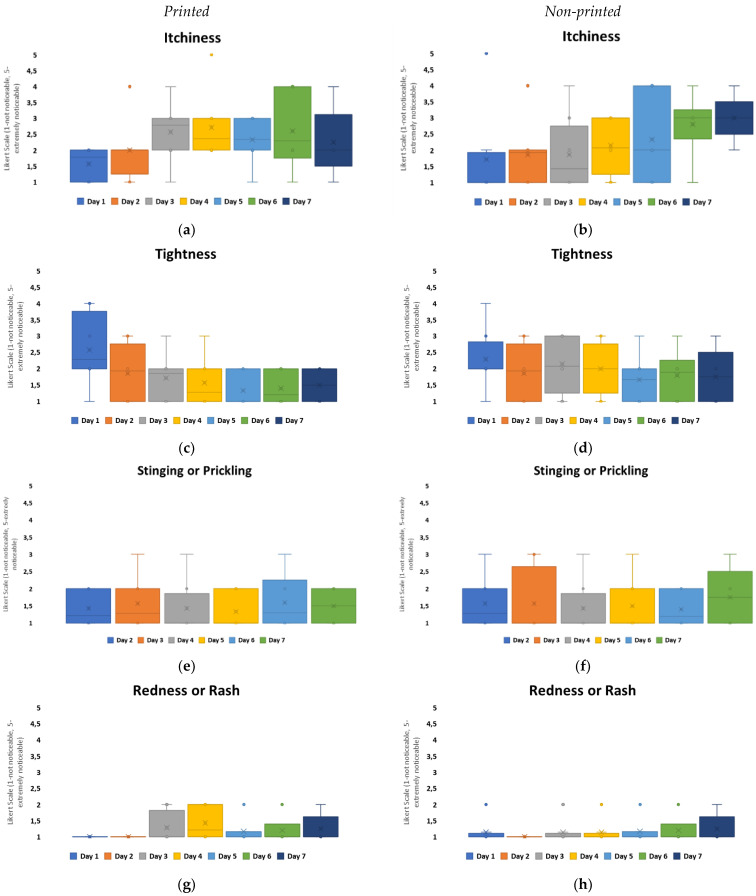
Box plots to show user perception for comfort and wear for printed (left column) and non-printed (right column) design configurations over the 7-day timeframe: (**a**,**b**) itchiness, (**c**,**d**) tightness, (**e**,**f**) stinging or prickling, (**g**,**h**) redness or rash.

To further evaluate the experience of non-printed designs in comparison to printed designs, four questions were asked daily for wear and comfort: the level of perceived itchiness, tightness, stinging or prickling, and redness or rash. Group C, which had the highest loss for patch attachment over the 7 days, see Figure 5, and the highest MVTR of all the groups, indicated through user perception that itchiness was the predominant user perception within both configurations, with a redness or rash presence being the least noticeable, Figure 7. On day 1, the highest awareness observed was tightness, which reduced over the timeframe. Itchiness was observed to increase from day 3 onwards. Both configurations, when compared, are not significantly different for user perception. In this exploratory study, the area covered by the printed electronics does not hinder the skin-wearable design interaction for volunteer perception.

**Figure 8:**
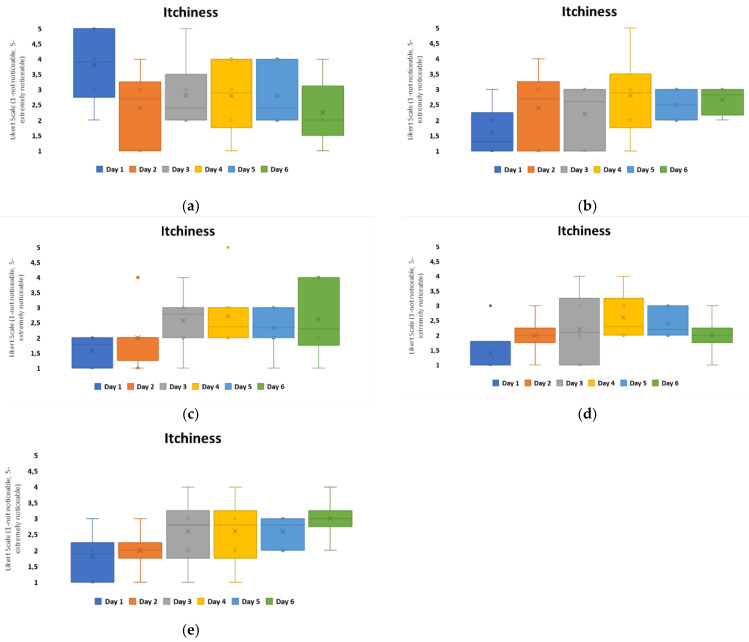
Box plots to show user perception for perceived itchiness for printed configurations over the 7-day timeframe: (**a**) Group A, (**b**) Group B, (**c**) Group C, (**d**) Group d, and (**e**) Group E.

To evaluate the level of itchiness experienced in relation to the patch material groups, printed configurations were studied further, Figure 8. The results indicate that Group A experienced the highest negative user perception on day 1. Group D had the lowest itchiness overall, with the user experience within this group remaining positive. For all other patch groups, the itchiness perception increased over the duration of the timeframe. Group C had the highest itchiness scores perceived by day 6 from all the groups. 

When relating these results to the material properties of the non-woven patches, Table 1, Group A, C, and D were different carrier polymers. Group A was an isotropic material, with the highest elasticity from the groups and the lowest hysteresis observed. Group C and D were anisotropic materials, with the highest hysteresis within the groups. The MVTR of these patch materials showed that Group A and D had similar MVTRs, 450–500 g/m^2^/24 h, and this is observed to correlate with decreased itchiness perceived by day 6. Group C, which had the highest MVTR, showed a general constantly perceived itchiness from day 2 onwards. The general consensus for improved wear and comfort within material developments has implied that higher MVTR numbers relate to improved comfort for users. This, however, is not the result observed in this study.

**Figure 9:**
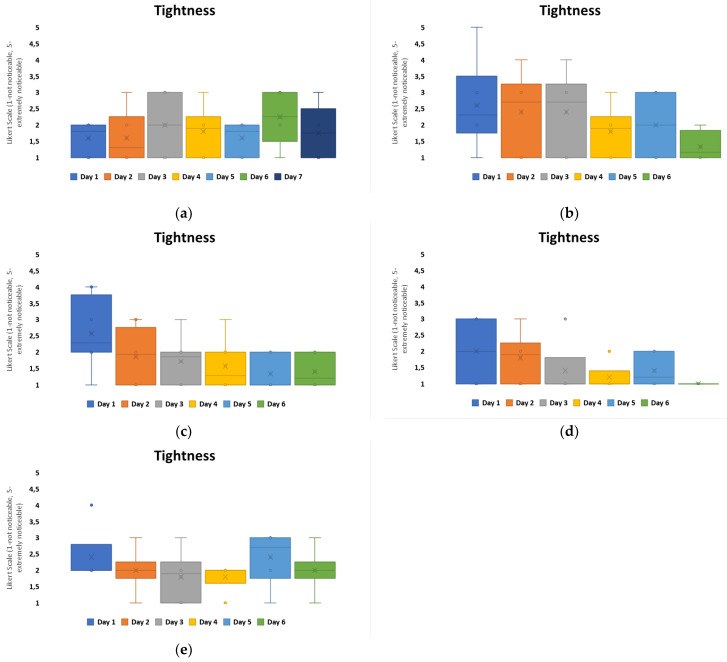
Box plots to show user perception for perceived tightness for printed configurations over the 7-day timeframe: (**a**) Group A, (**b**) Group B, (**c**) Group C, (**d**) Group d, and (**e**) Group E.

The results also compared tightness, which was the other noticeability marker that fluctuated overall within the configurations, Figure 7. Among all the groups, Group B was considered to have the highest level of negativity when it came to tightness, with a neutral-to-positive skew in perception present from day 4 onwards. Group C also showed a negative skew in perception on day 1 but from day 2 onwards, it remained neutral with a positive skew. The other groups’ tightness was perceived in a neutral to non-noticeable positive manner over the duration. Group D showed the lowest recorded noticeability, Figure 9. 

When comparing these results with material properties, Group B had the highest adhesive thickness, 100 microns, and isotropic behavior with high elasticity and low hysteresis. This appears to have a negative impact for user perception, if correlated with itchiness, Figure 8, as patches that performed more positively for user perception were anisotropic and had higher hysteresis levels.

### 3.4. Visual Observations

**Figure 10:**
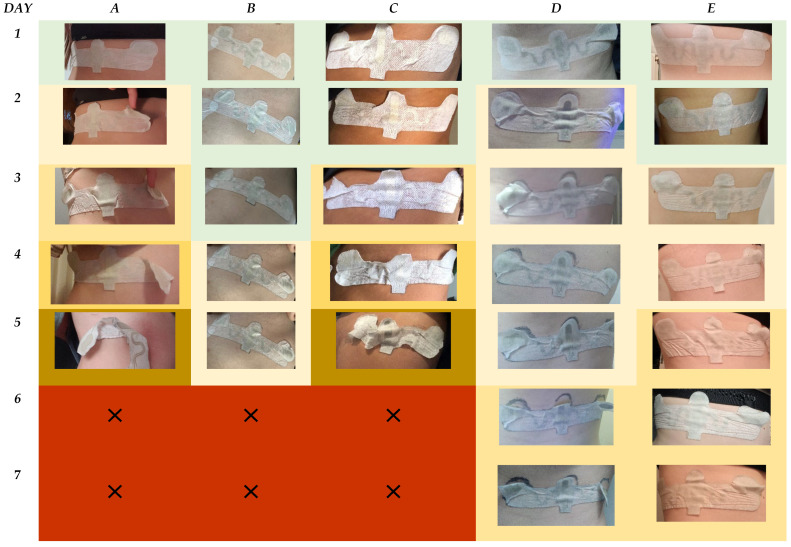
Images to show the worst-case scenario for the printed configuration of Groups A to E over the 7-day timeframe. The results are color-coded with green: intact device, orange: compromised device, and red: patch failure. The gradation in color for orange indicates an increased deterioration of the patch.

To compare user perception wear, comfort, and durability with the visual observation of skin–material interaction over the timeframe, printed configurations for the worst-case scenario of the groups are shown over the 7-day timeframe, Figure 10. 

The images show that Groups A, B, and C detached after day 5, whereas Groups D and E remained for all 7 days. All printed configurations showed failure within design underneath the printed substrate and commonly in large areas of the printed substrate where the sensors within the design are located on the edges. This is shown by delamination from the skin in the form of air pockets. In Groups A and C, the formation of these air pockets is quickly followed by full failure within the edges of the wearable design, with only the central part remaining intact with low adherence. 

For Groups B and D, dirt accumulation is observed from day 3 onwards. Group B had the highest adhesive thickness but different mechanical properties, with Group B being a TPU and D being a PES. Group D had a higher level of hysteresis compared to B and as such, in the images, it can be seen that the severity of dirt accumulation increased in comparison.

Group E is shown to have the highest integrity for patch–skin interaction. With failure observed on the edges, this could be related to external clothing that caused edge release within this location. 

All the printed configurations showed a similar trend for mismatch in delamination properties of the wearable devices. Whereby for areas of the design not covered by the printed substrate, the delamination occurs in regular horizontal stripes across the patch, most visualized in Group E.

**Figure 11:**
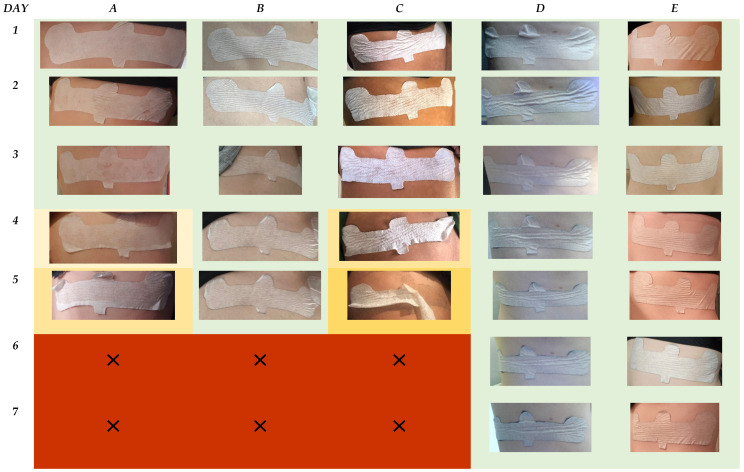
Images to show the worst-case scenario for the non-printed configuration of Groups A to E over the 7-day timeframe. The results are color-coded with green: intact device, orange: compromised device, and red: patch failure. The gradation in color for orange indicates an increased deterioration of the patch.

For comparison, the worst-case scenario for non-printed configurations of the groups were visualized (Figure 11). The results indicated that delamination for the groups occur within a re-occurring horizontal pattern over the wearable design. Groups A, B, and C were removed after day 5. For Groups A and B, the configuration appears to show high integrity with the skin on day 5; therefore, removal could correlate instead with the volunteer themselves for the perception of discomfort, which may have attributed to early removal. For Group C, however, the adhesion of the patch begins to mechanically fail from day 4 at the edges, which increases rapidly by day 5.

For Group D, as observed in the printed configuration, dirt accumulation begins to appear from day 2. The level of dirt accumulation in comparison to the printed configuration is lower over the duration of wear. For Groups D and E, adhesive failure is observed on the top edges of the wearable design, which could be attributed to external clothing impact. 

Within this study, external variables related to clothing of volunteers were not controlled. This was to provide a snapshot of what the general northern European population could wear and the impact this could have for wearable device placement. With increased activity through exercise, one of the main criteria for volunteer selection, accelerated testing for durability of material and placement of this wearable design was observed on volunteers. As such, the results indicate that for females, in comparison to males, this wearable design could be compromised due to the presence of undergarments. 

Overall, the results obtained from the photo diaries were significant in providing indications on which material and design properties attributed to device–skin failure.

## 4. Discussion

To enable the integration of medical wearables within the healthcare framework, three main parameters need to be optimized: high-resolution sensor signal acquisition, wearability, and comfort. Within wearable device developments, a large proportion of investigation and material optimization has focused on sensor signal optimization. Wear and comfort are integral for real-world application and user compliance, and as such, they should not be overlooked. Wearable medical device design has highlighted the use of more flexible, conformable form factors to improve user compliance and continuous monitoring over longer periods of time. Hybrid printed electronics (HPEs) is a methodology gaining increased traction for the development of new conformable wearable devices. Within this investigation, the impact of material properties from medical-grade non-woven tape selected within a wearable HPE design, and the relationship with printed electronic circuitry and sensor functionality, was conducted.

User perception and material integrity were evaluated using two different assessment tools: Likert scales and photo diaries. The resultant relationship between material properties and behavioral and mechanical implications was assessed over a 7-day timeframe. The volunteer group selected performed high-activity movements for a minimum of 2 h daily. This enabled insight into accelerated wear testing for mechanical failure of the wearable device from skin, as well as the impact of sweat and breathability beneath these wearable configurations. The location of the wearable devices, on the lower torso, was important for sensor signal acquisition in cardiovascular use–cases, where healthcare professionals have indicated the desired use of wearables within home settings.

Two configurations of the wearable design—one with printed electronics and one without—were applied onto volunteers, with five different non-woven tape groups (A to E). The non-woven tape groups were all commercial materials noted for longer-term wear and increased comfort and wearability within devices. However, the material composition for all groups differed, with carrier material either being TPU-based—which typically resulted in isotropic, low-residual-strain mechanical properties—or PET- and PES-based material—which resulted in anisotropic, high-residual-strain mechanical properties. The groups selected had significant differences in MVTR, a parameter generally related in the literature to improved comfort, and adhesive thickness, which is generally chosen for improved adherence. 

The results from this study, however, indicate that a more selective process for medical non-woven tapes within the wearable device build-up is needed for application on the lower torso. The relationship between increased comfort and higher MVTR was not observed, whereas the Likert scale results showed that a higher level of itchiness was observed, with MVTR of 1200 g/m^2^/24 h in comparison to 400 g/m^2^/24 h. The adhesive thickness also did not indicate increased wearable-skin integrity over the duration of wear. The patch group with the highest adhesive thickness, 100 microns, had a 20% loss in adherence from day 5 in comparison to the lowest adhesive thickness, 51 microns, which remained for all 7 days for all volunteers. 

The results also do not indicate that a lower MVTR and lower adhesive thickness relate to longer wear time. Instead, behavioral characteristics collected via Likert scales and observation assessment via photo diaries showed contradicting and complex trends. 

For user perception overall, both printed and non-printed configurations performed similar in relation to overall experience. This is with printed configurations performing worse on days 1 and 2 but both reaching the same neutrality and positive user perception with increased time. The use of the Likert scale questions did show that when the user perception was subjectively divided into specific questions, user perception for printed to non-printed configurations varied. 

On a daily basis, volunteers were asked for the perception of itchiness, tightness, redness, and pimples. When taking into account the baseline volunteer questionnaire results before application, any negative indications from these volunteers can be correlated with the patch configuration, as the majority of volunteers did not show any previous negative perceptions for their skin. From the four markers assessed daily for comfort of the configurations, tightness and itchiness were more negatively perceived within the questionnaire. When relating these results to the material properties of the patch groups, groups with low hysteresis despite high elasticity were shown to have increased negative perceptions compared to higher-hysteresis groups. 

The similar user perception for patch integrity of both printed and non-printed configurations shown by volunteers did not correspond to patch–skin interaction observed within the photo diaries of volunteers. Printed and non-printed configurations were shown to mechanically fail in adhesion to the skin in different ways. Printed configurations delaminated below the printed substrate within the design, starting on the edges of the printed design from day 2 onwards, whereas within non-printed configurations, delamination occurred in repetitive horizontal patterns over the wearable design. Failure also consistently occurred for both designs from the outer edges of the upper half of the patch. This can also relate to the impact of external clothing causing higher stress on those locations. 

Overall, the results are significant for indicating failure modes within the wearable design. The material itself is not shown to undergo breakdown during wear; rather, it is the adhesive–skin interaction and adhesive–wearable patch interaction that are seen to become compromised. The pressure-sensitive acrylic adhesive within the groups indicate failure within the cohesive properties of the adhesive to skin, as seen with dirt accumulation in Groups C and D, as the viscoelastic properties of the adhesive fail to match the elasticity of the skin with increased wear. The adhesive strength of the PSA, for adhesive-to-skin interaction, was observed through the complete delamination of wearable patches in Groups A, B, and C from day 6. The ability to compare printed and non-printed configurations enables increased learning for the significance of adhesive coverage within a wearable patch. Failure was visually more significant within printed configurations in comparison to non-printed configurations. 

As well as adhesive coverage, lower breathability below the printed substrate of the design caused a significant impact on device–skin integrity, causing bubbling beneath those surfaces. It is observed as the most prominent in Group C, which had the highest MVTR in comparison to the other groups. The higher mismatch in breathability beneath the skin appears to have detrimental results for patch–skin integrity and could also be associated with the increased awareness of volunteers. 

Non-printed configuration delamination is seen to relate strongly to the body itself. On the lower torso, the direction of Langer lines for skin tension are within a horizontal repetitive pattern that matches the delamination pattern seen in this configuration with increased time. These observations are key for development of new wearable designs that are dependent on body location and the varied elasticity and deformation of skin present for longer wear times. 

## 5. Conclusions

Overall, the complexity within this study highlights the significance non-woven tape selection has on the successful application of medical wearables. Both mechanical material and design impact the durability, wear, and comfort for wearable device build-up. The results indicate directions for improved wearable design for both user perception as well as reduction in critical failure zones for longer-term wear in home settings.

The complexity indicated within current wearable device design highlights the need for further work, both to improve user perception and for longer-term wear possibilities for the future development of large-area electronic skins.

As small volunteer groups were used within this exploratory study, increasing the group size—and adding the body location and age group as control groups—could further confirm and improve the user perception of comfort and wearability, as well as provide significant learning for the development of longer-term medical wearables.

## Figures and Tables

**Table 1:** Non-woven tape material properties divided into Groups A to E.

Patch Group	Adhesive Thickness (Micron)	MVTR (g/m^2^/24 h)	Carrier Material
A	75	500	PU
B	100	350	PU
C	75	1200	PES
D	75	450	PET
E	51	400	PU

## Data Availability

No new data were created or analyzed in this study. Data sharing is not applicable to this article.
